# Multi-Marker Evaluation of Creatinine, Cystatin C and β2-Microglobulin for GFR Estimation in Stage 3–4 CKD Using the 2021 CKD-EPI Equations

**DOI:** 10.3390/ijms27020862

**Published:** 2026-01-15

**Authors:** Nurulamin Abu Bakar, Nurul Izzati Hamzan, Siti Nurwani Ahmad Ridzuan, Izatus Shima Taib, Zariyantey Abdul Hamid, Anasufiza Habib, Noor Hafizah Hassan

**Affiliations:** 1 Centre of Diagnostics, Therapeutics and Investigative Studies (CODTIS), Faculty of Health Sciences, Universiti Kebangsaan Malaysia, Jalan Raja Muda Abdul Aziz, Kuala Lumpur 50300, Malaysia; izatusshima@ukm.edu.my (I.S.T.); zyantey@ukm.edu.my (Z.A.H.); 2 Special Protein Unit, Specialized Diagnostic Centre, Institute for Medical Research, National Institutes of Health, Jalan Pahang, Kuala Lumpur 50588, Malaysia; nurulizzati.h@moh.gov.my (N.I.H.); siti.nurwani@moh.gov.my (S.N.A.R.); 3 Specialized Diagnostic Centre, Institute for Medical Research, National Institutes of Health, Jalan Pahang, Kuala Lumpur 50588, Malaysia; anasufiza@moh.gov.my

**Keywords:** chronic kidney disease (CKD), stage 3–4 CKD, glomerular filtration rate (GFR), 2021 CKD-EPI equations, serum creatinine, cystatin C, β2-microglobulin (β2M), molecular biomarkers, diagnostic accuracy, ROC curve

## Abstract

Chronic kidney disease (CKD) is a progressive disease in which accurate estimation of glomerular filtration rate (GFR) is essential for staging and guiding therapy. Serum creatinine is widely used but influenced by non-renal factors, while cystatin C and β2-microglobulin (β2M) may provide complementary information related to filtration and tubular or inflammatory factors. This study compared the discriminatory performance of creatinine, cystatin C and β2M for separating CKD stage 3 from stage 4 within the 2021 CKD-EPI eGFR framework in 45 adults with CKD stages 3–4. CKD stage classification was defined using the 2021 CKD-EPI creatinine and creatinine–cystatin C equations (eGFRcr, eGFRcr–cys) with a threshold of 30 mL/min/1.73 m^2^. Receiver operating characteristic (ROC) analysis evaluated each marker’s ability to distinguish moderate from severe CKD. Creatinine showed high diagnostic accuracy (AUC up to 0.98). Cystatin C achieved 100% specificity at the optimal cut-off for severe CKD and showed comparable diagnostic accuracy to creatinine under the eGFRcr–cys framework (AUC 0.978 vs. 0.957). β2M demonstrated AUCs up to 0.97, with sensitivity and specificity above 90%. These findings support a multi-marker evaluation within the 2021 CKD-EPI-based staging, rather than validation against measured GFR. Larger studies incorporating measured GFR and relevant clinical confounders are warranted.

## 1. Introduction

Chronic kidney disease (CKD) is a major global health problem, affecting nearly 10% of the adult population and contributing substantially to cardiovascular morbidity, premature mortality and healthcare costs [1]. The progressive and often irreversible decline in kidney function in CKD is closely linked to structural damage, including glomerulosclerosis, tubulointerstitial fibrosis and microvascular injury. Accurate assessment of glomerular filtration rate (GFR) is therefore essential not only for staging CKD, but also for risk stratification, prognostication and timely initiation of nephroprotective therapies [2].

GFR is widely recognized as the best overall indicator of kidney function. Direct measurement of GFR using exogenous filtration markers such as inulin, iohexol or radioisotopes provides high accuracy, but these methods are complex, time-consuming, expensive and not suitable for routine clinical practice [3,4,5]. As a result, most clinical decisions rely on estimated GFR (eGFR) derived from endogenous serum biomarkers. Over the past decades, several creatinine-based equations have been developed, culminating in the recent 2021 CKD-EPI equations, which removed race coefficients to improve equity and reduce bias in GFR estimation [6].

Serum creatinine remains the most commonly used endogenous filtration marker because it is inexpensive, widely available and well-integrated into clinical guidelines. However, creatinine levels are strongly influenced by non-renal factors such as muscle mass, diet, age and sex, and may be less reliable at extremes of body composition or in certain ethnic groups [6]. These limitations have led to growing interest in alternative or complementary biomarkers that more closely reflect kidney function and underlying pathophysiology.

Cystatin C is a low-molecular-weight protein produced at a relatively constant rate by all nucleated cells. It is freely filtered by the glomeruli and almost completely reabsorbed and catabolized in the proximal tubules, with minimal extrarenal elimination. Because its concentration is less affected by muscle mass and diet, cystatin C has been proposed as a more specific marker of GFR, particularly in populations where creatinine may be misleading [7,8]. Incorporation of cystatin C into eGFR equations, as in the 2021 CKD-EPI creatinine–cystatin C (eGFRcr–cys) equation, has been shown to improve precision and reduce bias in GFR estimation.

Beta-2 microglobulin (β2M) is another low-molecular-weight protein that is filtered by the glomeruli and almost completely reabsorbed in the proximal tubules under normal conditions. Elevated β2M concentrations can reflect both reduced glomerular filtration and tubulointerstitial injury, and have been associated with CKD progression, inflammation and cardiovascular risk [9,10,11]. These characteristics suggest that β2M may provide complementary pathophysiological information to creatinine and cystatin C, particularly in patients with moderate to severe CKD, where structural damage is more advanced. However, β2M has been less extensively studied as a routine marker for GFR estimation, and its role within contemporary eGFR frameworks remains to be clarified.

Taken together, these observations support a multi-marker approach that integrates creatinine, cystatin C and β2M to capture different molecular and physiological aspects of kidney dysfunction. Such an approach may refine GFR estimation, improve discrimination between stages of CKD and better align with our evolving understanding of the molecular mechanisms underlying kidney disease. The aim of this study was to compare the diagnostic performance of serum creatinine, cystatin C and β2M for discriminating CKD stage 3 from stage 4 when classification was defined using the 2021 CKD-EPI creatinine (eGFRcr) and creatinine–cystatin C (eGFRcr–cys) equations in adults with CKD stages 3–4. Specifically, we evaluated their ability to distinguish moderate from severe CKD at a clinically relevant threshold of 30 mL/min/1.73 m^2^ and examined β2-microglobulin as a complementary biomarker whose interpretation may reflect both filtration impairment and tubular factors.

## 2. Results

Baseline demographic and laboratory characteristics of the study cohort are provided in Appendix A (Table A1), including age, sex, reciprocal biomarker values (1/cystatin C, 1/creatinine and 1/β2M), and eGFR values calculated using the 2021 CKD-EPI equations (eGFRcr and eGFRcr–cys). Because this study involved secondary analysis of archived, de-identified laboratory data, clinical information such as primary kidney disease etiology and comorbidities (e.g., diabetes mellitus and hypertension) was not consistently available and therefore was not analyzed.

### 2.1. Relationship Between Serum Cystatin C, Creatinine, and β2M with GFR

To evaluate the relationship between each biomarker and estimated GFR, we calculated Pearson correlation coefficients (*r*) using the reciprocal values of serum creatinine, cystatin C, and β2M. Estimated GFR based on serum creatinine (eGFRcr) showed a very strong correlation with reciprocal serum creatinine (*r* = 0.906; *n* = 45), and strong correlations with reciprocal serum cystatin C (*r* = 0.775; *n* = 45) and reciprocal serum β2M (*r* = 0.836; *n* = 45). The corresponding coefficients of determination (*R^2^*) were 0.821, 0.601, and 0.699, respectively (Table 1A).

Estimated GFR based on the combined serum creatinine–cystatin C equation (eGFRcr–cys) also demonstrated strong linear relationships with all three biomarkers. Reciprocal serum creatinine correlated strongly with eGFRcr–cys (*r* = 0.806; *R*^2^ = 0.651; *n* = 45), while reciprocal serum cystatin C (*r* = 0.960; *R*^2^ = 0.922) and reciprocal serum β2M (*r* = 0.944; *R*^2^ = 0.892) showed very strong correlations (Table 1B).

**Figure 1:**
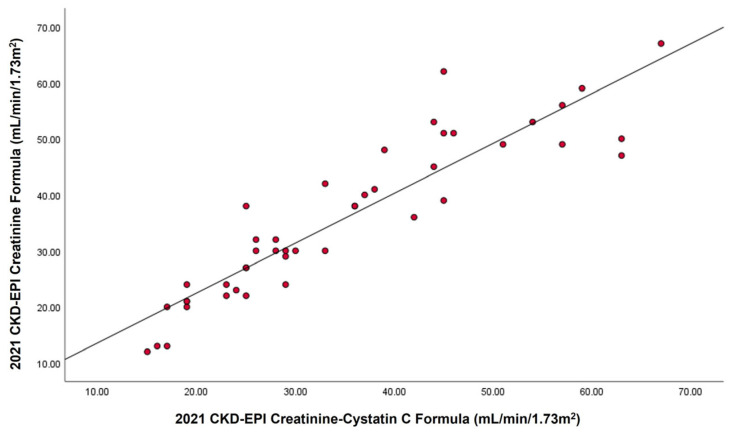
Scatter plot showing the relationship between estimated glomerular filtration rate based on the 2021 CKD-EPI creatinine equation (eGFRcr) and the 2021 CKD-EPI creatinine–cystatin C equation (eGFRcr–cys) in 45 adults with CKD stages 3–4. The solid line represents the line of best fit with the corresponding Pearson correlation coefficient (*r*).

Additionally, eGFRcr showed a strong correlation (r = 0.914; *n* = 45) with eGFRcr–cys, as shown in Figure 1.

### 2.2. Diagnostic Accuracy Comparison Between Serum Creatinine, Cystatin C and β2M

**Figure 2:**
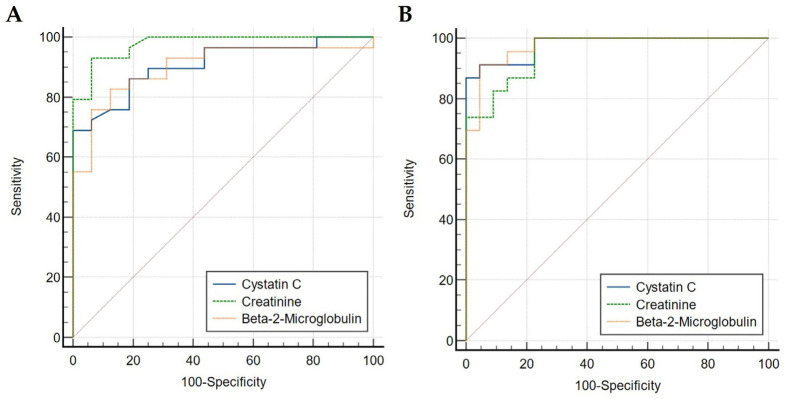
Receiver operating characteristic (ROC) curves for serum creatinine, cystatin C and β2-microglobulin (β2M) in discriminating moderate (CKD stage 3) from severe (CKD stage 4) kidney dysfunction at an eGFR threshold of 30 mL/min/1.73 m^2^. (**A**) ROC curves based on the 2021 CKD-EPI creatinine equation (eGFRcr). (**B**) ROC curves based on the 2021 CKD-EPI creatinine–cystatin C equation (eGFRcr–cys). The area under the curve (AUC) and 95% confidence intervals for each biomarker are shown.

The diagnostic performance of serum creatinine, cystatin C, and β2M was assessed using ROC curve analysis at a predefined eGFR threshold of 30 mL/min per 1.73 m^2^ to distinguish between moderate (CKD stage 3) and severe (CKD stage 4) kidney dysfunction. Using the 2021 CKD-EPI creatinine equation (eGFRcr), ROC analysis showed that the area under the curve (AUC) for serum creatinine [AUC = 0.977 (95% CI: 0.882–0.999)] was comparable to that of serum cystatin C (*p* = 0.1035) [AUC = 0.908 (95% CI: 0.784–0.974)] and serum β2M (*p* = 0.1003) [AUC = 0.901 (95% CI: 0.775–0.970)] (Figure 2A).

When the 2021 CKD-EPI creatinine–cystatin C equation (eGFRcr–cys) was applied, the ROC curves again demonstrated similarly high diagnostic accuracy for all three markers. The AUC for serum creatinine [AUC = 0.957 (95% CI: 0.850–0.995)] was comparable to that of serum cystatin C (*p* = 0.4295) [AUC = 0.978 (95% CI: 0.883–0.999)] and serum β2M (*p* = 0.5465) [AUC = 0.974 (95% CI: 0.877–0.999)] (Figure 2B).

At the predefined cut-off of 30 mL/min per 1.73 m^2^, the sensitivity, specificity, positive predictive value (PPV), negative predictive value (NPV), and Youden Index for each GFR marker were determined.

Using the 2021 CKD-EPI Creatinine Formula (eGFRcr), serum creatinine showed a sensitivity of 93.1%, specificity of 93.75%, PPV of 94.8%, and NPV of 91.3%, with a Youden Index of 0.87. Serum cystatin C demonstrated a sensitivity of 68.97%, specificity of 100%, PPV of 100%, and NPV of 78.6%, with a Youden Index of 0.69. Serum β2M exhibited a sensitivity of 82.76%, specificity of 87.50%, PPV of 85.2%, and NPV of 85.1%, with a Youden Index of 0.70 (Table 2A).

When the 2021 CKD-EPI creatinine–cystatin C equation (eGFRcr–cys) was used, serum creatinine achieved a sensitivity of 100%, specificity of 77.27%, PPV of 80.6%, and NPV of 100%, with a Youden Index of 0.77. Serum cystatin C showed a sensitivity of 86.96%, specificity of 100%, PPV of 100%, and NPV of 84.0%, with a Youden Index of 0.87. Serum β2M demonstrated a sensitivity of 91.30%, specificity of 95.45%, PPV of 95.2%, and NPV of 91.7%, with a Youden Index of 0.87 (Table 2B).

## 3. Discussion

Chronic kidney disease (CKD) is a major health burden, and accurate estimation of glomerular filtration rate (GFR) remains central to staging and clinical decision-making [1,2,3]. Creatinine-based eGFR is widely used but is influenced by non-GFR determinants, supporting interest in complementary biomarkers such as cystatin C and other low-molecular-weight proteins [5]. In this study, we compared creatinine, cystatin C and β2-microglobulin (β2M) within the 2021 CKD-EPI eGFR framework to inform a pragmatic multi-marker approach for discriminating CKD stage 3 from stage 4.

In this context, our findings reinforce the diagnostic robustness of creatinine when interpreted with the 2021 CKD-EPI creatinine equation (eGFRcr), showing high sensitivity and specificity in differentiating between CKD stages 3 and 4. Previous local work comparing the MDRD and CKD-EPI equations against isotopic GFR methods in Malaysian patients confirmed the superiority of CKD-EPI, further supporting its use in this setting [12]. Taken together, these data indicate that creatinine-based eGFR remains a reliable foundation for CKD staging when used with contemporary equations, although its limitations must still be acknowledged in specific clinical scenarios.

Cystatin C demonstrated superior specificity (100%) and higher predictive accuracy at the critical threshold of 30 mL/min/1.73 m^2^ in our study. This underscores cystatin C’s clinical utility as a confirmatory marker for advanced kidney dysfunction, particularly in cases where creatinine-based estimates may be misleading. Biologically, cystatin C is a low-molecular-weight cysteine protease inhibitor produced at a relatively constant rate by all nucleated cells and freely filtered by the glomeruli, with almost complete reabsorption and catabolism in the proximal tubules. Its serum concentration is therefore less affected by muscle mass and may provide a reflection less dependent on muscle mass interpreted cautiously as a marker of glomerular filtration than creatinine. Our results align with multicenter studies demonstrating cystatin C’s added value in risk prediction beyond creatinine and with evaluations showing its clinical utility and cost-effectiveness in primary care CKD management [13,14]. Together, these findings suggest that cystatin C could improve diagnostic precision and optimize resource allocation in CKD care, particularly when used in a targeted manner for patients with uncertain or borderline creatinine-based eGFR.

Importantly, this study also highlights the diagnostic value of beta-2 microglobulin (β2M). While not yet widely used in routine nephrology practice, β2M demonstrated balanced sensitivity and specificity, particularly under the creatinine–cystatin C framework (eGFRcr–cys), supporting its potential as a complementary biomarker. β2M is a component of the major histocompatibility complex class I molecule and is a low-molecular-weight protein that is freely filtered by the glomerulus and normally reabsorbed and catabolized by proximal tubular cells. Consequently, circulating β2M may reflect a combination of reduced filtration and altered tubular handling. In CKD, tubulointerstitial injury may reduce proximal tubular uptake and metabolism, potentially amplifying β2M elevations beyond what is expected from GFR decline alone. This dual dependence highlights why β2M should be interpreted cautiously as a filtration marker and supports its potential role as a biomarker capturing both filtration impairment and tubular involvement [15].

From a translational perspective, β2M can also be influenced by non-GFR determinants and extrarenal factors, including systemic inflammation, immune activation, and malignancy, which may affect generalizability and clinical implementation [16]. Nevertheless, its association with the severity of kidney injury, including acute kidney injury, supports its broader relevance as a complementary biomarker in renal disease [10].

Therefore, in current practice, β2M is best positioned as a complementary biomarker and a candidate component for future multi-marker equations rather than a standalone filtration marker. Future studies should incorporate inflammatory indices and relevant comorbidities, evaluate assay standardization and cost, and compare performance against measured GFR and emerging multi-marker equations incorporating β2M and β-trace protein [11].

Several studies in Asia have evaluated the 2021 CKD-EPI equation. For example, Pakistani and Korean cohorts have assessed its performance in creatinine-based GFR estimation and its impact on CKD prevalence and risk prediction [17,18]. In China, the ES-CKD study validated a β2M-based GFR equation and compared it with cystatin C-inclusive equations, demonstrating improved accuracy with multi-marker approaches [19]. A large multi-country analysis further showed variable effects of the 2021 CKD-EPI equation across Asian populations [20]. Our study extends this body of work by evaluating a three-marker panel (creatinine, cystatin C and β2M) within the 2021 CKD-EPI equations in a Malaysian CKD cohort, providing novel regional data and supporting the feasibility of a multi-marker strategy in this population.

The integration of multiple biomarkers, particularly creatinine, cystatin C and β2M, reflects a broader movement toward multi-marker strategies in CKD biology and diagnosis. Such approaches aim to capture different molecular and physiological pathways involved in kidney injury, including altered glomerular filtration, proximal tubular reabsorption and chronic inflammation. In principle, this may improve staging accuracy, inform risk prediction for cardiovascular complications and guide earlier therapeutic interventions. This is particularly relevant given the close interplay between CKD, chronic inflammation and cardiovascular disease, which remains the leading cause of mortality among patients with end-stage kidney disease [3]. By combining filtration-based and inflammation-linked markers, our findings align with the emerging paradigm of molecular diagnostics emphasized in this Special Issue, where biochemical markers serve as accessible surrogates of underlying molecular mechanisms.

A limitation of this study is the relatively small cohort (*n* = 45), which restricts generalizability and limits the precision of some estimates. Because the study population was limited to CKD stages 3–4, the findings may not extrapolate to earlier CKD (stages 1–2), dialysis-dependent kidney failure, or other Asian populations with different comorbidity patterns and body composition. Validation across a broader CKD spectrum and ethnically diverse cohorts, ideally against measured GFR, will be important to define the clinical contexts in which cystatin C and β2M add the greatest value.

Nevertheless, this represents one of the first Malaysian datasets evaluating the 2021 CKD-EPI equations in combination with cystatin C and β2M, offering preliminary insights into their clinical performance in a real-world population. Another important limitation is that GFR was not directly measured using an exogenous filtration marker. Instead, we used eGFR derived from the 2021 CKD-EPI creatinine and creatinine–cystatin C equations as the reference. As a result, the strong performance of creatinine and cystatin C partly reflects their incorporation into these equations, and our findings should be interpreted as an evaluation of marker performance within the CKD-EPI framework rather than an independent validation against measured GFR. Larger multicenter studies are needed to validate these findings and to assess additional emerging markers such as β-trace protein, which, when combined with β2M, may yield even greater diagnostic accuracy across diverse ethnic groups without reliance on race-based adjustments [11]. Cost-effectiveness studies will also be essential to support broader adoption of cystatin C and β2M in routine practice, particularly in resource-constrained healthcare systems [14].

Our findings highlight that while serum creatinine remains a reliable primary marker for GFR estimation, cystatin C provides superior specificity in identifying severe CKD, and β2M offers balanced diagnostic performance as a complementary marker. From a biological perspective, cystatin C and β2M not only reflect renal filtration but are also associated with systemic processes such as inflammation and cardiovascular risk, underscoring their relevance beyond kidney function alone [10,11]. Diagnostic integration of these markers, particularly within the 2021 CKD-EPI equations, improves accuracy at critical thresholds and may reduce reliance on creatinine alone, which is influenced by extrarenal factors. Clinically, adopting a multi-marker strategy could facilitate earlier identification of patients at higher risk of CKD progression and related complications, enabling timely interventions such as tighter blood pressure and metabolic control, avoidance of nephrotoxic drugs and earlier referral for nephrology care. These therapeutic implications are especially relevant in Malaysia and other regions with rising CKD prevalence, where cost-effectiveness and accessibility remain critical considerations [1,2,3,14]. By linking biochemical markers to underlying pathophysiological processes, this multi-marker approach illustrates how molecularly informed diagnostics can support more precise and personalized care in CKD, in line with the objectives of this Special Issue.

## 4. Materials and Methods

### 4.1. Patients’ Samples

This retrospective observational study was based on the analysis of laboratory data from 45 patients (22 males and 23 females; aged 25–75 years) with chronic kidney disease (CKD) stages 3 to 4 who attended the Nephrology Clinic at the Institute of Urology and Nephrology, Kuala Lumpur Hospital (Appendix A). All data were obtained from routine clinical testing. The study was conducted in accordance with the Declaration of Helsinki and was approved by the Institutional Review Board of Ministry of Health’s Medical Research Ethics Committee (MREC) [NMRR ID-25-02170-XEB (IIR)].

### 4.2. Cystatin C, Beta-2 Microglobulin (β2M) and Creatinine Assays

Serum cystatin C and β2M levels were measured using a particle-enhanced nephelometric assay (PENIA) on a Siemens nephelometer system (Siemens Healthcare Diagnostics, Forchheim, Germany). Serum creatinine was measured using the modified Jaffe method on a Roche chemistry analyzer (Roche Diagnostics, Mannheim, Germany).

These three markers were selected because they represent complementary aspects of CKD pathophysiology, reflecting glomerular filtration (creatinine and cystatin C) and low-molecular-weight protein handling (β2M).

### 4.3. GFR Estimation

The estimated glomerular filtration rate (eGFR) was calculated using the 2021 CKD-EPI equations for creatinine and creatinine–cystatin C:

#### 4.3.1. 2021 CKD-EPI Creatinine Formula (eGFRcr)

The creatinine-based eGFR (eGFRcr) was calculated using the following formula:

eGFRcr (mL/min/1.73 m^2^) = 142 × (Scr/A) − B × 0.9938Age × (1.012 if female)

where Scr is serum creatinine (mg/dL); A and B depend on sex and creatinine level:
**Sex****Creatinine Level****A****B**Female≤0.7 mg/dL0.7−0.241Female>0.7 mg/dL0.7−1.200Male≤0.9 mg/dL0.9−0.302Male>0.9 mg/dL0.9−1.200

#### 4.3.2. 2021 CKD-EPI Creatinine–Cystatin C Formula (eGFRcr–cys)

The creatinine–cystatin C-based eGFR (eGFRcr–cys) was calculated as:

eGFRcr-cys (mL/min/1.73 m^2^) = 135 × (Scr/A) − B × (Scys/C) − D × 0.9938Age × (1.012 if female)

where Scr is serum creatinine (mg/dL) and Scys is serum cystatin C (mg/L); A, B, C and D depend on sex and levels of creatinine and cystatin C:
**Sex****Creatinine Level****Cystatin C Level****A****B****C****D**Female≤0.7 mg/dL≤0.8 mg/L0.7−0.2190.8−0.323Female≤0.7 mg/dL>0.8 mg/L0.7−0.2190.8−0.778Female>0.7 mg/dL≤0.8 mg/L0.7−0.5440.8−0.323Female>0.7 mg/dL>0.8 mg/L0.7−0.5440.8−0.778Male≤0.9 mg/dL≤0.8 mg/L0.9−0.1440.8−0.323Male≤0.9 mg/dL>0.8 mg/L0.9−0.1440.8−0.778Male>0.9 mg/dL≤0.8 mg/L0.9−0.5440.8−0.323Male>0.9 mg/dL>0.8 mg/L0.9−0.5440.8−0.778

### 4.4. Statistical Methods

#### 4.4.1. Correlation Analysis

The relationship between serum creatinine, cystatin C, and β2M with estimated GFR was evaluated using correlation analysis of their reciprocal values to linearize the curvilinear relationship with eGFRcr and eGFRcr–cys. Correlation analysis was also performed to assess the association between eGFRcr and eGFRcr–cys. Pearson correlation coefficients (*r*) and coefficient of determination (*R*^2^) were calculated, with statistical significance defined as *p* < 0.05. All analyses were performed using SPSS for Windows, version 29.0.1.0 (IBM Corp., Armonk, NY, USA).

#### 4.4.2. Receiver Operating Curve (ROC) Analysis

The diagnostic accuracy of reciprocal serum cystatin C, serum creatinine and serum β2M was assessed using ROC curve analysis to discriminate between patients with moderate (CKD stage 3) and severe (CKD stage 4) kidney dysfunction. A predefined eGFR threshold of 30 mL/min/1.73 m^2^ was applied to classify kidney function.

This study did not validate biomarkers against directly measured GFR. CKD stage classification for CKD stage 3 and CKD stage 4 was based on the 2021 CKD-EPI equations (eGFRcr and eGFRcr–cys) using a predefined threshold of 30 mL/min/1.73 m^2^. Because creatinine and cystatin C are components of these equations, their apparent discriminatory performance may be inflated relative to an independent reference standard. Therefore, the findings should be interpreted as a comparative assessment within the CKD-EPI framework.

ROC curves were generated by plotting sensitivity against 1—specificity. The area under the curve (AUC) and 95% confidence intervals (CIs) were calculated using the binomial exact method. Sensitivity, specificity, positive predictive value (PPV), and negative predictive value (NPV) were determined, with the optimal cut-off value identified using the Youden Index. Comparisons between AUCs of different GFR markers were performed using the DeLong method. Statistical significance was defined as *p* < 0.05. All analyses were conducted using MedCalc for Windows, version 23.09 (MedCalc Software Ltd., Ostend, Belgium).

## 5. Conclusions

In adults with CKD stages 3–4, serum creatinine, cystatin C and β2-microglobulin demonstrated high discriminatory performance for separating stage 3 from stage 4 kidney dysfunction when classification was defined using the 2021 CKD-EPI equations, including eGFRcr and eGFRcr–cys. Cystatin C showed high specificity at the 30 mL/min/1.73 m^2^ threshold, while β2-microglobulin provided a balanced sensitivity and specificity profile, supporting its potential role as a complementary biomarker. These findings should be interpreted as a comparative assessment within CKD-EPI-based staging rather than a validation against measured GFR. Larger studies incorporating measured GFR, broader CKD spectra and relevant clinical confounders are warranted.

## Figures and Tables

**Table 1:** (**A**). Correlation between reciprocal serum creatinine, cystatin C and β2-microglobulin (β2M) and estimated glomerular filtration rate based on the 2021 CKD-EPI creatinine equation (eGFRcr). Pearson correlation coefficients (*r*) and coefficients of determination (*R*^2^) are shown (*n* = 45). (**B**). Correlation between reciprocal serum creatinine, cystatin C and β2-microglobulin (β2M) and estimated glomerular filtration rate based on the 2021 CKD-EPI creatinine–cystatin C equation (eGFRcr–cys). Pearson correlation coefficients (*r*) and coefficients of determination (*R*^2^) are shown (*n* = 45).

(**A**)			
**GFR Marker**	***r***	***R*** **^2^**	**Linear Equation**
Serum creatinine	0.906	0.821	y = (1.48 × 10^−3^) + (1.21 × 10^−4^)x
Serum cystatin C	0.775	0.601	y = (0.2 × 10^−3^) + (9.5 × 10^−3^)x
Serum β2M	0.836	0.699	y = 0.02 + (6.38 × 10^−3^)x
(**B**)			
**GFR Marker**	***r***	***R*** **^2^**	**Linear Equation**
Serum creatinine	0.806	0.651	y = (2.15 × 10^−3^) + (1.05 × 10^−4^)x
Serum cystatin C	0.960	0.922	y = 0.13 + (0.01)x
Serum β2M	0.944	0.892	y = (7.8 × 10^−3^) + 7.01 × (10^−3^)x

**Table 2:** (**A**). Diagnostic performance of serum creatinine, cystatin C and β2-microglobulin (β2M) for discriminating moderate (CKD stage 3) from severe (CKD stage 4) kidney dysfunction using the 2021 CKD-EPI creatinine equation (eGFRcr) at an eGFR threshold of 30 mL/min/1.73 m^2^. Sensitivity, specificity, positive predictive value (PPV), negative predictive value (NPV) and Youden Index are presented. (**B**). Diagnostic performance of serum creatinine, cystatin C and β2-microglobulin (β2M) for discriminating moderate (CKD stage 3) from severe (CKD stage 4) kidney dysfunction using the 2021 CKD-EPI creatinine–cystatin C equation (eGFRcr–cys) at an eGFR threshold of 30 mL/min/1.73 m^2^. Sensitivity, specificity, positive predictive value (PPV), negative predictive value (NPV) and Youden Index are presented.

(**A**)					
**GFR Marker**	**Sensitivity** **(%)**	**Specificity** **(%)**	**PPV** **(%)**	**NPV** **(%)**	**Youden Index**
Serum creatinine	93.10	93.75	94.80	91.30	0.87
Serum cystatin C	68.97	100.00	100.00	78.60	0.69
Serum β2M	82.76	87.50	85.20	85.10	0.70
(**B**)					
**GFR Marker**	**Sensitivity** **(%)**	**Specificity** **(%)**	**PPV** **(%)**	**NPV** **(%)**	**Youden Index**
Serum creatinine	100.00	77.27	80.60	100.00	0.77
Serum cystatin C	86.96	100.00	100.00	84.00	0.87
Serum β2M	91.30	95.45	95.20	91.70	0.87

## Data Availability

The original contributions presented in this study are included in the article and Appendix A. Further inquiries can be directed to the corresponding authors.

## Abbreviations

The following abbreviations are used in this manuscript:

AUC	Area under the curve
CI	confidence intervals
CKD	Chronic kidney disease
CKD-EPI	Chronic Kidney Disease Epidemiology Collaboration
eGFR	Estimated glomerular filtration rate
eGFRcr	The 2021 CKD-EPI creatinine equation
eGFRcr–cys	The 2021 CKD-EPI creatinine–cystatin C equation
GFR	Glomerular filtration rate
KDIGO	The Kidney Disease: Improving Global Outcomes
KDOQI	The National Kidney Foundation–Kidney Disease Outcomes Quality Initiative
MDRD	Modification of Diet in Renal Disease
NPV	negative predictive value
*p*	*p*-value
PENIA	particle-enhanced nephelometric assay
PPV	positive predictive value
*r*	Pearson correlation coefficients
*R*^2^	Coefficient of determination
ROC	Receiver operating characteristic
β2M	Beta-2 microglobulin

## Appendix A

**Table A1:** Baseline demographic and laboratory characteristics of the study cohort (*n* = 45).

Patient ID	Age	Sex	1/Cystatin C	1/Creatinine	1/β2M	eGFRcr	eGFRcr–cys
P1	75	Male	0.35461	0.00369	0.135869565	20	19
P2	51	Male	0.757576	0.0075758	0.395256917	56	57
P3	47	Female	0.383142	0.0027701	0.132802125	13	17
P4	53	Male	0.934579	0.0068966	0.396825397	50	63
P5	56	Male	0.389105	0.0038023	0.18115942	24	23
P6	68	Male	0.44843	0.0065359	0.235849057	42	33
P7	59	Female	0.350877	0.0027855	0.10940919	12	15
P8	40	Male	0.332226	0.0030864	0.149476831	21	19
P9	42	Female	0.47619	0.0042553	0.191204589	22	25
P10	56	Male	0.395257	0.0045662	0.182481752	30	26
P11	50	Female	0.460829	0.0056497	0.199203187	30	30
P12	61	Male	0.471698	0.0046948	0.240963855	30	29
P13	50	Male	0.409836	0.0034722	0.129366106	22	23
P14	70	Female	0.332226	0.0052632	0.104166667	24	19
P15	45	Male	0.952381	0.0063694	0.469483568	47	63
P16	67	Male	0.485437	0.0047393	0.273972603	29	29
P17	45	Male	0.502513	0.0064103	0.244498778	48	39
P18	51	Female	0.649351	0.008	0.334448161	45	44
P19	60	Male	0.355872	0.0023585	0.130718954	13	16
P20	27	Female	0.421941	0.0045662	0.17452007	27	25
P21	54	Female	0.561798	0.0073529	0.280898876	40	37
P22	65	Female	0.793651	0.009901	0.41322314	53	54
P23	25	Female	0.340136	0.0060241	0.082644628	38	25
P24	74	Female	0.485437	0.0064516	0.183823529	30	28
P25	56	Female	0.558659	0.0071942	0.275482094	38	36
P26	59	Male	0.60241	0.0072993	0.3003003	51	45
P27	31	Female	0.442478	0.0040816	0.192307692	23	24
P28	50	Male	0.609756	0.0068966	0.421940928	51	46
P29	32	Female	0.552486	0.009434	0.354609929	62	45
P30	70	Female	0.4329	0.0057143	0.207900208	27	25
P31	27	Male	0.740741	0.006993	0.381679389	59	59
P32	50	Male	0.714286	0.0066667	0.338983051	49	51
P33	38	Female	0.869565	0.0104167	0.487804878	67	67
P34	33	Female	0.299401	0.0037594	0.149700599	20	17
P35	47	Male	0.526316	0.0053191	0.25974026	38	36
P36	50	Male	0.571429	0.0071429	0.256410256	53	44
P37	46	Female	0.564972	0.0071942	0.249376559	41	38
P38	65	Female	0.746269	0.0076923	0.272479564	39	45
P39	57	Female	0.581395	0.005848	0.289017341	30	33
P40	66	Female	0.408163	0.0064935	0.160771704	32	26
P41	66	Male	0.847458	0.0072464	0.46728972	49	57
P42	60	Male	0.52356	0.0039526	0.236966825	24	29
P43	38	Female	0.440529	0.0056818	0.215053763	32	28
P44	58	Male	0.671141	0.0054054	0.318471338	36	42
P45	56	Female	0.357143	0.0044248	0.154083205	21	19
